# Surgery combined with anlotinib for local control of patients with resectable extremity desmoid fibromatosis: a retrospective study

**DOI:** 10.3389/fphar.2024.1357071

**Published:** 2024-03-07

**Authors:** Dechao Yuan, Yong Liu, Xiang Fang, Fan Wu, Senlin Lei, Linqi Tu, Fuguo Kuang, Yawei Gou, Chunfu Gong, Wenli Zhang, Hong Duan

**Affiliations:** ^1^ Department of Orthopedics, Orthopedic Research Institute, West China Hospital, Sichuan University, Chengdu, China; ^2^ Department of Burn and Plastic Surgery, West China Hospital, Sichuan University, Chengdu, China; ^3^ Department of Orthopedics, People’s Fourth Hospital of Sichuan Province, Chengdu, China

**Keywords:** desmoid fibromatosis, surgery, anlotinib, local recurrence, side effects

## Abstract

**Background:** Desmoid fibromatosis (DF) is a pathological intermediate fibroblastoma that is difficult to control locally due to its invasive nature, especially in the extremities. Although anlotinib demonstrated efficacy in treating DF with tolerable safety, the impact of surgical intervention in conjunction with anlotinib administration on local control in patients with extremity DF remains undetermined.

**Methods:** We conducted a retrospective examination of the clinical medical documentation belonging to patients with resectable DF of the extremities who were treated with surgery between January 2010 and June 2022. The patients were divided into two cohorts: surgery alone cohort and surgery combined with anlotinib group (surgery plus anlotinib cohort), crossover to surgery plus anlotinib cohort was admissible for patients in the surgery alone cohort who experienced disease recurrence postoperatively. Clinical data such as basic information, tumor location, anlotinib toxicity, time to recurrence, surgical complications, follow-up time, visual analogue scale (VAS) score and Musculoskeletal Tumor Society (MSTS) score at the last follow-up were collected.

**Results:** In total, 48 consecutive patients (19 males and 29 females) with resectable DF of the extremities, including 25 patients in the surgery alone cohort, 23 patients in the surgery plus anlotinib cohort, and 10 patients who were transferred from the surgery alone cohort to the surgery plus anlotinib cohort. The VAS score at the last follow-up was 5 (IQR, 3–6) in the surgery alone cohort and 2 (IQR, 1–3) in the surgery plus anlotinib cohort, respectively; the MSTS score at the last follow-up was 19 (IQR, 16.5–24) in the surgery alone cohort and 27 (IQR, 25–28) in the surgery plus anlotinib cohort, respectively; these characteristics were statistically different between the two cohorts. The 3-year recurrence-free survival (RFS) of the surgery alone cohort and the surgery plus anlotinib cohort were 37.7% and 72.6%, respectively, and the difference was statistically significant (*p* = 0.022).

**Conclusion:** Surgery combined with anlotinib appears to be effective in controlling local recurrence in patients with resectable DF of the extremities, and the side effects were acceptable.

## 1 Introduction

Desmoid fibromatosis (DF) is a pathologically intermediate fibroblastic tumor that is difficult to control locally due to its infiltrative nature. DF consists of spindle-shaped cells embedded within a dense collagenous matrix abundantly interspersed with prominent blood vessels, which often demonstrating a locally infiltrative proliferation pattern. DF possesses an annual incidence of 5–6 cases per million inhabitants, which may be underestimated due to the stealthiness and spontaneous regression of the disease (Kasper et al., 2015; Kasper et al., 2017a; Penel et al., 2021). DF possesses the potential to appear anywhere throughout the body, mainly in the extremities, intra-abdominal, and abdominal wall. On the contrary, extremity DF generally predicts an increased risk of recurrence and a poor prognosis because tumors are often adjacent to vascular nerves (Wirth et al., 2018; Mandel et al., 2022; Lehnhardt et al., 2023).

DF are predominantly sporadic, and approximately 90% of DF are associated with mutations in exon 3 of the somatic b-catenin gene (CTNNB1). However, although CTNNB1 plays a role in the pathogenesis of DF, the prognostic value of CTNNB1 mutations has yet to be elucidated thus far (Penel et al., 2022). Ten percent of DF are associated with germline adenomatous polyposis mutations and familial adenomatous polyposis (Norkowski et al., 2020; Riedel and Agulnik, 2022). In addition, it has been posited that estrogenic hormones may be implicated in the pathogenic genesis of DF, such that the incidence of DF is highest in women during or after pregnancy (Trautmann et al., 2020; Riedel and Agulnik, 2022).

Two decades earlier, surgical resection with negative margins was deemed the archetypal intervention for patients with DF. However, due to the high local recurrence and complications after surgery, a transition to a more conservative approach has been newly promulgated. An international guideline for the management of DF has recently been introduced, which takes into account the patient’s perspective. Active surveillance with scheduled magnetic resonance imaging (MRI) is first-line treatment for DF. The anatomic location of the tumor should be considered before any therapeutic intervention is identified and risk-benefit assessments should be performed, weighing adverse effects against lasting sequelae (Shido et al., 2009; Prodinger et al., 2013; Dürr et al., 2020; Sobczuk et al., 2021).

For progressive DF in the extremities, medical treatment is recommended following active surveillance. Medical treatment includes surgery, radiation therapy, low-dose or conventional chemotherapy, non-steroidal anti-inflammatory drugs (NSAIDs), anti-hormone therapy, and tyrosine kinase inhibitors (TKIs), but there is no standard treatment regimen (Prendergast et al., 2022; Tsukamoto et al., 2023). To date, there is no evidence to confirm the efficacy of NSAIDs and anti-hormone therapy for patients with DF according to the Desmoid Working Group (Kasper et al., 2015; Kasper et al., 2017a). TKIs, including anlotinib, sorafenib, imatinib, and pazopanib, have been evaluated as new non-chemotherapeutic systemic therapies in patients with unresectable, recurrent, or progressive DF and have yielded some promising clinical results (Gounder et al., 2011; Kasper et al., 2017b; Agresta et al., 2018; Zheng et al., 2020). Anlotinib is a novel multi-targeted TKI that selectively inhibited platelet-derived growth factor receptor, vascular endothelial growth factor receptor-1, -2, -3, and hepatic cytokine receptor (Shen et al., 2018). However, the effect of surgical treatment combined with anlotinib on local control in patients with extremity DF remains undetermined; therefore, we retrospectively compared the clinical efficacy of surgery alone and surgery combined with anlotinib. To evaluate the local control rate of surgical treatment combined with anlotinib.

## 2 Patients and methods

### 2.1 Study population and design

We conducted a retrospective examination of the clinical medical documentation belonging to patients with resectable DF of the extremities who were treated with surgery between January 2010 and June 2022 at our center. The criteria requisite for inclusion in the study were delineated as follows: 1) The diagnosis of DF was pathologically confirmed in the Department of Pathology of West China Hospital; 2) tumor located in the extremity, including buttock; 3) patients had clinical symptoms, mainly including pain, functional limitation, and compression symptoms, which were not relieved after 6 months of observation; 4) the tumor could be surgically removed without damaging vital neurovascular bundle; 5)tumor resection was performed by Professor Hong Duan; and 6) postoperative follow-up time greater than 12 months. The exclusion criteria from the study were delineated as follows: 1) chemotherapy or radiation therapy had been used before treatment in our hospital; 2) the patient’s general condition was poor and could not tolerate surgery; 3) the patient’s follow-up data were insufficient.

**FIGURE 1:**
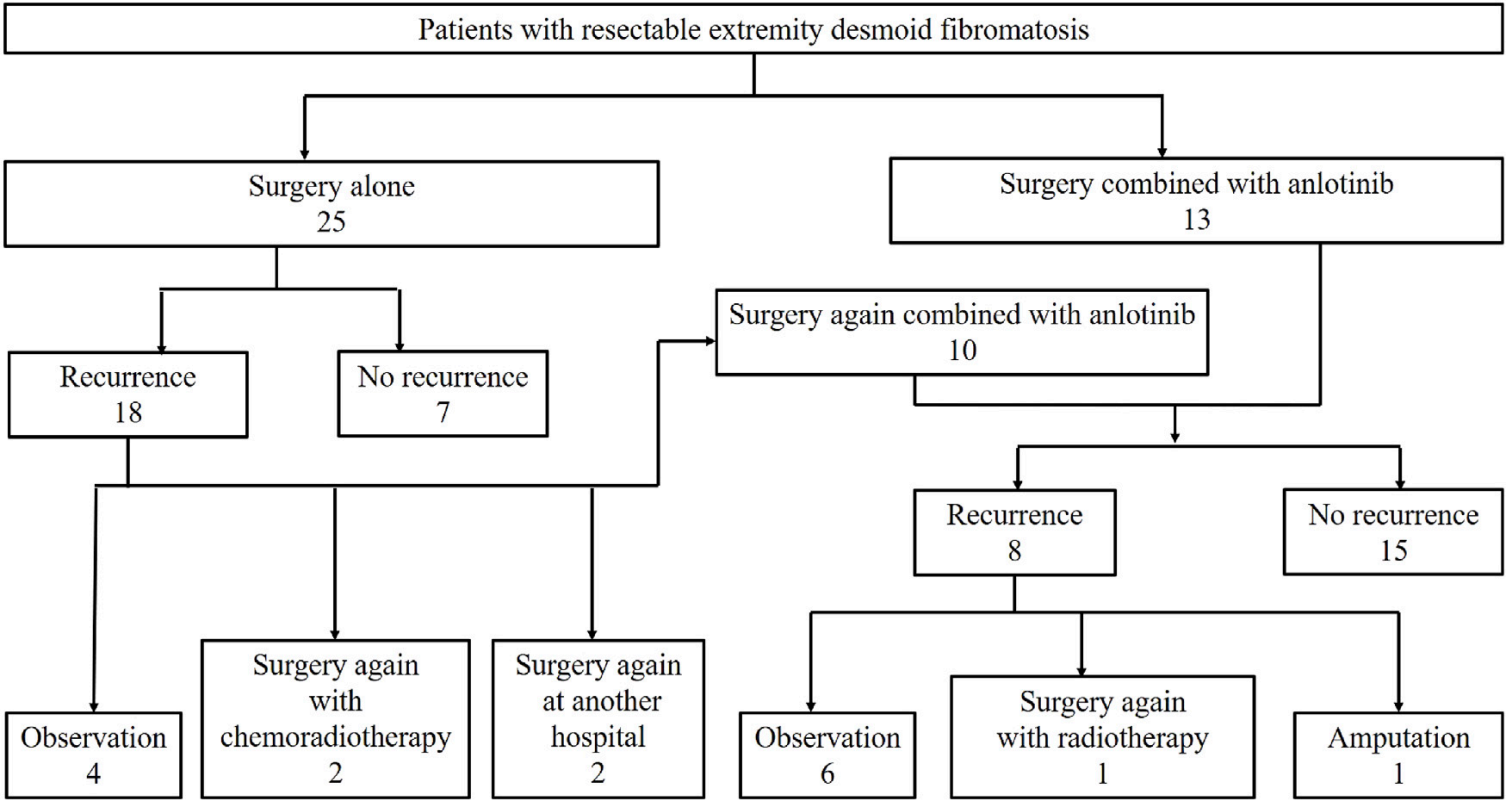
Flow chart of patient treatment strategy and results.

The patients were divided into two cohorts: surgery alone cohort and surgery combined with anlotinib group (surgery plus anlotinib cohort), as shown in Figure 1. Before 2018, we treated DF mainly by surgery with or without chemoradiotherapy. The treatment strategy after 2018 was surgery combined with anlotinib if the patient had clinical symptoms that did not relieve after 6 months of observation. Crossover to the surgery plus anlotinib cohort was admissible for patients in the surgery alone cohort who experienced disease recurrence postoperatively.

Resectable tumor was delineated as: marginal or extensive resection of the tumor was considered feasible without injury to the vital neurovascular bundle, or would engender tolerable morbidity subsequent to extensive or marginal resection. Whenever possible, the aim was to obtain a negative resection margin, unless the tumor was adjacent to the neurovascular bundle. For cases where negative margins were difficult to obtain, marginal resection was chosen. The treatment strategy and the final decision on whether to operate or otherwise were discussed at a multidisciplinary oncology meeting. All patients taking anlotinib provided informed consent for anlotinib therapy. This study was presented to and approved by the Institutional Review Board of Sichuan University West China Hospital (No 2022793).

### 2.2 Clinical data collection of patients

Routine clinical and imaging examinations were performed at monthly outpatient follow-up after surgery, and MRI examinations were performed at the third, sixth and 12th month, and then once a year thereafter. During follow-up, local tumor recurrence was found, and the patient was asked to return to the hospital for continued treatment in Hong Duan’s treatment group. If the patient found abnormalities (mass, pain, functional limitations, etc.), the frequency of MRI examinations increased. Recurrence was assessed by MRI. The subsequent clinical information was assembled: age, gender, status of disease, therapeutic history, tumor size, tumor location, date of surgery, toxicity of anlotinib, time to recurrence, operative complications, length of follow-up, visual analogue scale (VAS) score and Musculoskeletal Tumor Society (MSTS) score at the last follow-up, date of death if available. The size of the DF was defined as the maximum diameter on MRI prior to surgery or anlotinib therapy.

### 2.3 The use of anlotinib

For patients in the surgery plus anlotinib cohort, the starting dose of anlotinib was 8 mg once a day for 2 weeks of treatment followed by the cessation of treatment for 1 week. Preoperative and postoperative medication should be used for at least 4 courses, respectively. Postoperative medication should be prolonged to 1 year as far as possible, and the medication regimen and specific time of postoperative withdrawal should be comprehensively determined according to whether the patient had recurrence and adverse reactions. The dose was reduced to 6 mg if the patient experienced intolerable or uncontrolled pharmaceutical-induced toxicity. If a patient had relapsed following surgery combined with anlotinib and was observed with progressive disease or clinical symptoms, the dose will be increased to 10 mg (Eisenhauer et al., 2009); if a patient developed refractory adverse reactions during the subsequent two cycles of 10 mg, the drug would be permanently discontinued and other treatment approaches would be employed.

### 2.4 Clinical evaluation

Recurrence-free survival (RFS) constituted the primary outcome measure and was defined as the time interval from the date of surgical intervention to the date of tumor recurrence or patient death due to the tumor or the last follow-up. Clinical efficacy was mainly evaluated by pain relief and functional activity, quantified by the VAS score and the MSTS score, respectively. Pharmaceutical-associated adverse effects were categorized and stratified according to the Common Terminology Criteria for Adverse Events (version 4.0) (Basch et al., 2021).

### 2.5 Data analysis

Differences between two cohorts of patients were evaluated using the Fisher’s exact test or chi-square or Mann-Whitney U test. Descriptive statistics included median, interquartile range (IQR), counts, and percentages. Kaplan-Meier survival analysis and log-rank test were used to compare the RFS and survival curves between the two cohorts, respectively. A *p*-value <0.05 was considered statistically significant. Statistical analysis was performed using IBM SPSS version 21.0 (IBM Corp.).

## 3 Results

### 3.1 Characteristics of the study population

From January 2010 to June 2022, 48 consecutive patients (19 males and 29 females) with resectable DF of the extremities were admitted, including 25 patients in the surgery alone cohort, 23 patients in the surgery plus anlotinib cohort, and 10 patients who were transferred from the surgery alone cohort to the surgery plus anlotinib cohort (Figure 1). The median age was 25 years (IQR, 19–38.8); the median tumor size was 8.2 (IQR, 5.8–11.3); the median number of previous tumor surgery was 2 (IQR, 1–2); there were 8 primary tumors and 40 recurrent tumors; the most common tumor location was the gluteal region, followed by the thigh and scapula region; these characteristics were not statistically different between the two cohorts. 13 patients underwent surgery before 2018 and 35 patients underwent surgery after 2018, of which all patients in the surgery plus anlotinib cohort underwent surgery after 2018, with a statistically significant difference between the two cohorts (Table 1).

**TABLE 1:** The particulars of the individuals included in this study at baseline.

Variables	Total (n = 48, %)	Number of patients (n, %)		*p*-value
		Surgery alone (n = 25)	Surgery with anlotinib (n = 23)	
Age at diagnosis (years)				0.269
Median	25	32	22	
IQR	19–38.8	19.5–42.5	18.0–33.0	
Sex				0.769
Male	19 (39.6)	9 (36.0)	10 (43.5)	
Female	29 (60.4)	16 (64.0)	13 (56.5)	
Tumor location				0.361
Gluteal region	12 (25.0)	5 (20.0)	7 (30.4)	
Thigh	10 (20.8)	5 (20.0)	5 (21.7)	
Scapular region	10 (20.8)	5 (20.0)	5 (21.7)	
Popliteal region	4 (8.3)	2 (8.0)	2 (8.7)	
Forearm	4 (8.3)	3 (12.0)	1 (4.3)	
Foot	4 (8.3)	3 (12.0)	1 (4.3)	
calf	3 (6.3)	2 (8.0)	1 (4.3)	
Axillary region	1 (2.1)	0 (0)	1 (4.3)	
Tumor size (cm)				0.635
Median	8.2	9.3	7.8	
IQR	5.8–11.3	5.8–13.2	5.9–11.2	
Number of previous tumor surgery				0.330
Median	2	2	1	
IQR	1–2	1–2	1–2	
Year of surgery				<0.001
2010–2017	13 (27.1)	13 (52.0)	0 (0)	
2018–2023	35 (72.9)	12 (48.0)	23 (100)	
Status of disease				0.303
Primary	8 (16.7)	3 (12.0)	5 (21.7)	
Recurrent	40 (83.3)	22 (88.0)	18 (78.3)	

IQR, interquartile range.

### 3.2 Treatment outcomes

The median follow-up time was 45 months (IQR, 28.5–66.5 months) in the surgery alone cohort and 40 months (IQR, 27–50 months) in the surgery plus anlotinib cohort; the median interval between surgery and recurrence was 17.5 months (IQR, 12.5–31 months) in the surgery alone cohort and 24 months (IQR, 19.5–35 months) in the surgery plus anlotinib cohort; these characteristics were not statistically different between the two cohorts (Table 2). The number of recurrences at the last follow-up was 18 and 8 in the surgery alone cohort and surgery plus anlotinib cohort, respectively, with a statistically significant difference between the two cohorts. Overall, none of the patients died from the disease. The VAS score at the last follow-up was 5 (IQR, 3–6) in the surgery alone cohort and 2 (IQR, 1–3) in the surgery plus anlotinib cohort, respectively; the MSTS score at the last follow-up was 19 (IQR, 16.5–24) in the surgery alone cohort and 27 (IQR, 25–28) in the surgery plus anlotinib cohort, respectively; these characteristics were statistically different between the two cohorts (Table 2).

**TABLE 2:** Treatment characteristics and outcome of the patients included in this study.

Variables	Number of patients (n, %)		*p*-value
	Surgery alone (n = 25)	Surgery with anlotinib (n = 23)	
Follow up period (months)			0.117
Median	45	40	
IQR	28.5–66.5	27–50	
Recurrences at the last follow-up			0.019
no	7 (28.0)	15 (65.2)	
yes	18 (72.0)	8 (34.8)	
Interval between the surgery and recurrence			0.165
Median	17.5	24	
IQR	12.5–31	19.5–35	
VAS score at the last follow-up			<0.001
Median	5	2	
IQR	3–6	1–3	
MSTS score at the last follow-up			<0.001
Median	19	27	
IQR	16.5–24	25–28	
The number of postoperative anlotinib treatment courses			-
Median	-	26	
IQR	-	21.5–42	
Surgical complications			0.748
Infection	1	2	
Wound healing issues	3	4	
Temporary iatrogenic nerve damage	2	1	

IQR, interquartile range.

**FIGURE 2:**
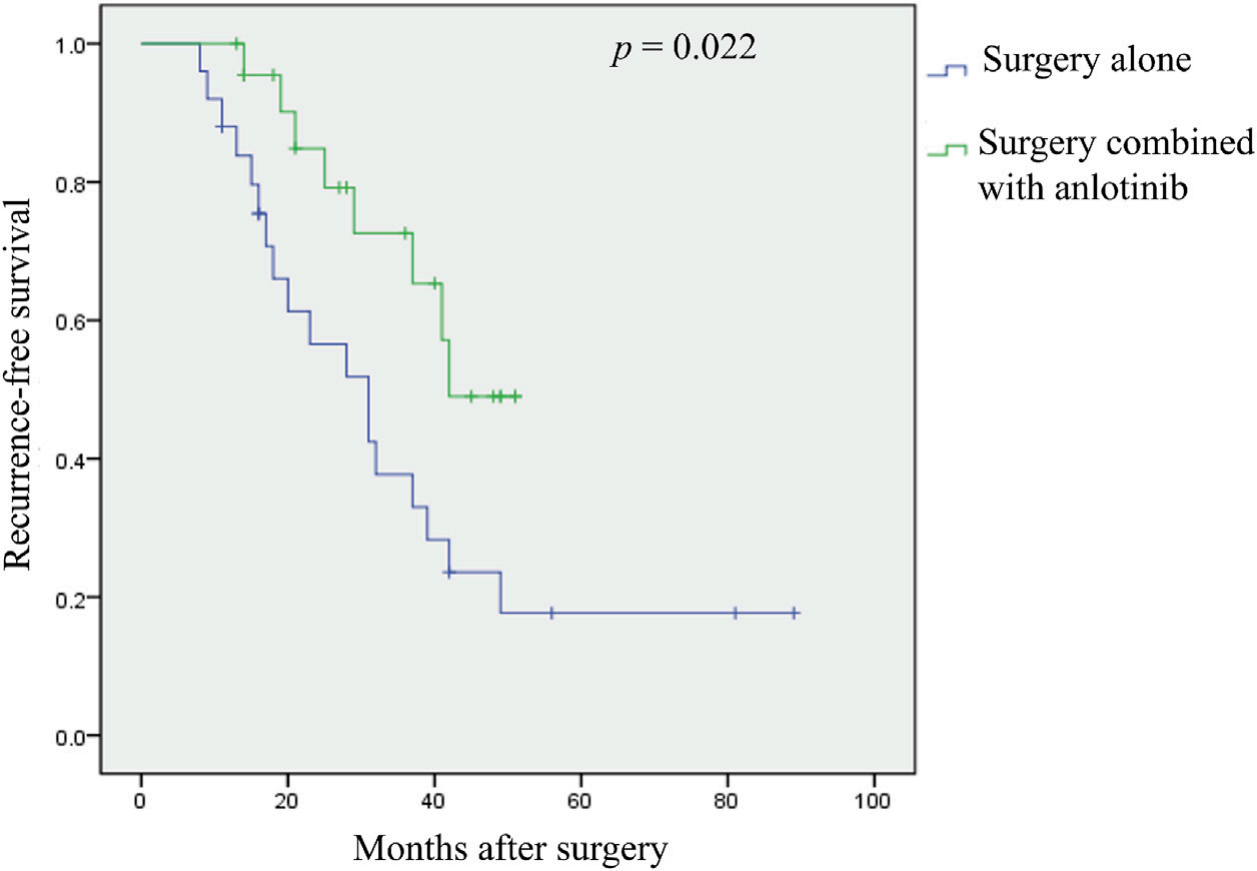
Kaplan-Meier analysis of recurrence—free survival was performed according to different treatment strategies.

**FIGURE 3:**
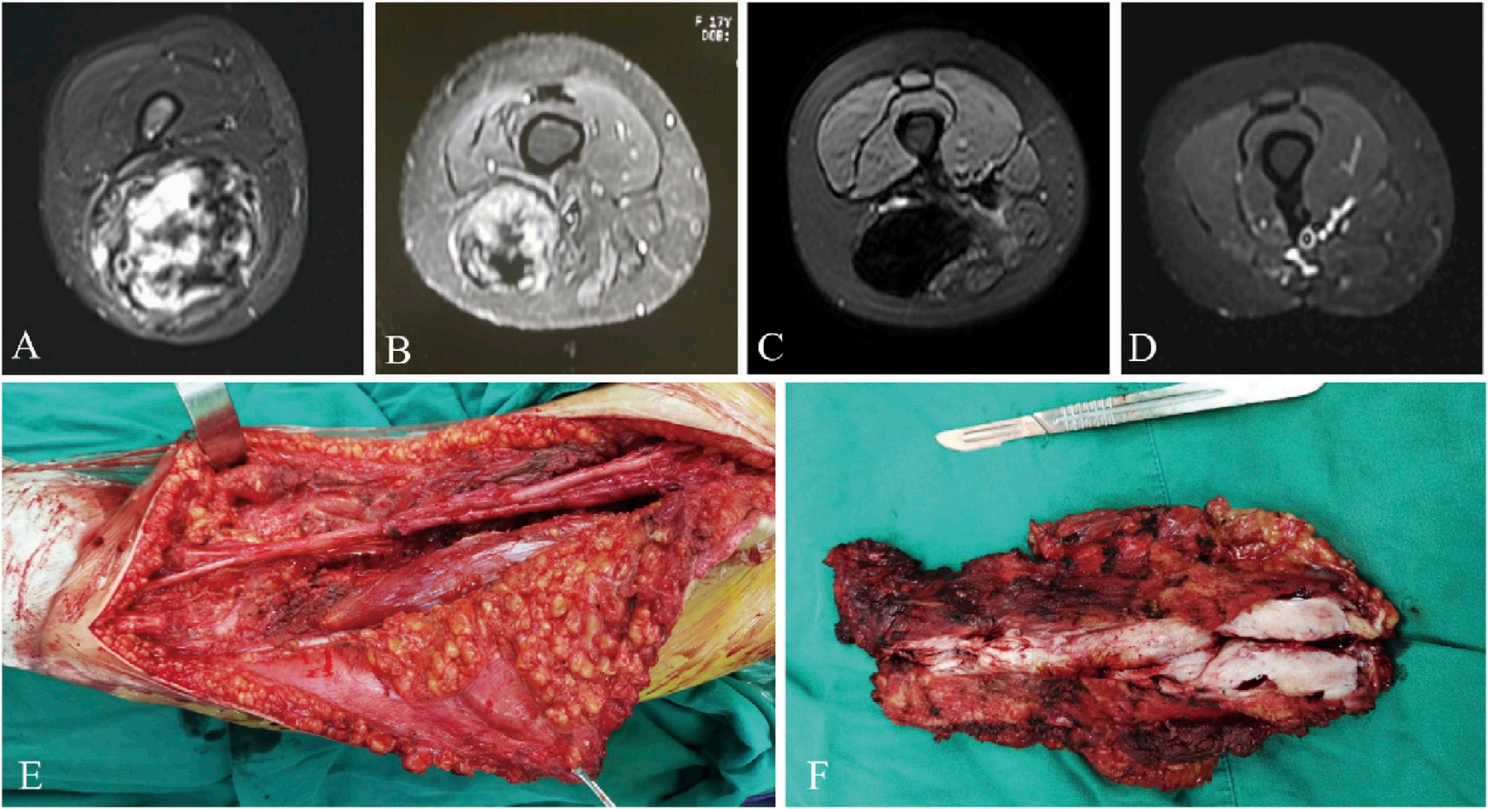
A 20-year-old female presented with postoperative recurrence of DF of right thigh **(A)**, which still recurred 17 months after tumor resection in our hospital **(B)**. MRI was re-examined after using anlotinib **(C)**, and the tumor was resected again with preserved vascular and nerve during the operation **(E, F)**, and MRI was re-performed 14 months after surgery **(D)**. MRI, magnetic resonance imaging.

**FIGURE 4:**
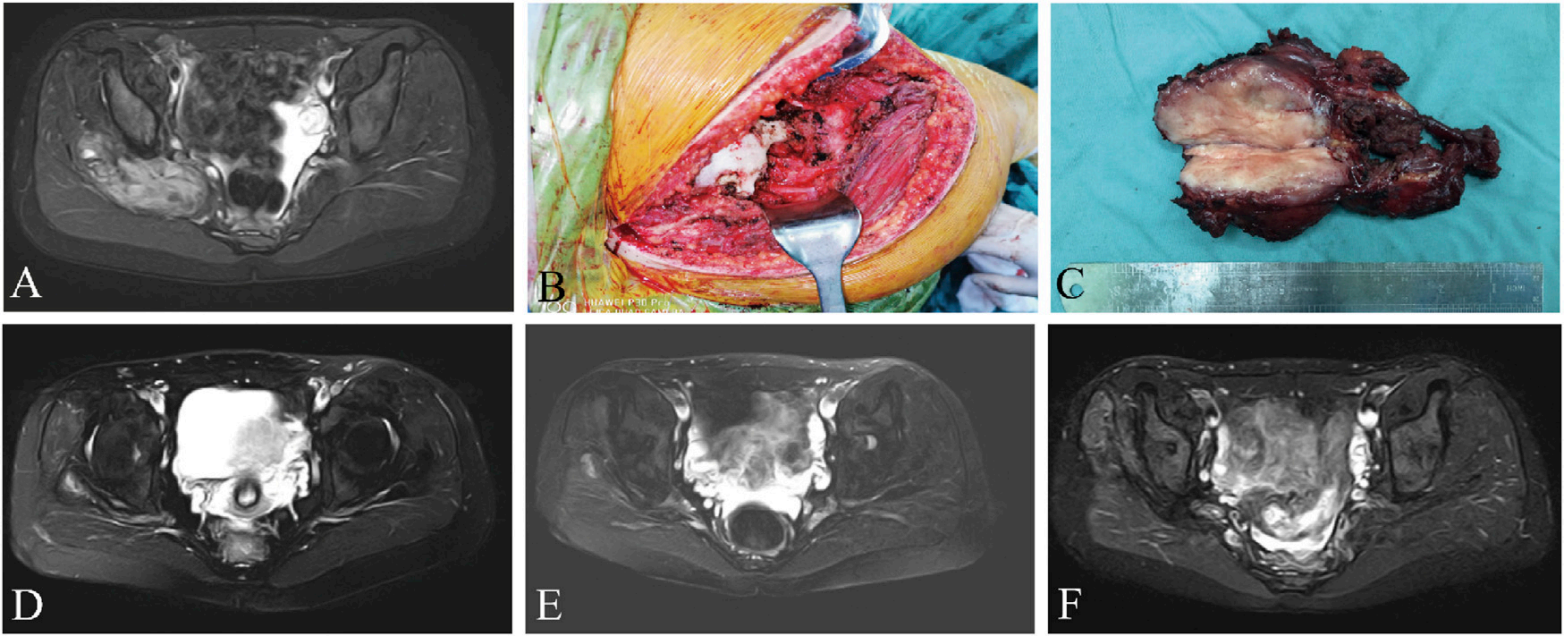
A 21-year-old female presented with DF of the right buttock, preoperative MRI **(A)**, surgical resection of the tumor **(B, C)**, and reexamination of MRI **(D–F)** after 27, 39, and 49 months after surgery, respectively. MRI, magnetic resonance imaging.

The median RFS was 31 months and the 3-year RFS rate was 37.7% in the surgery alone cohort; the median RFS was 42 months and the 3-year RFS rate was 72.6% in the surgery plus anlotinib cohort. Significant difference in RFS was observed between the two cohorts (*p* = 0.022, Figure 2). The typical case of the surgery alone cohort was shown in Figure 3: A 20-year-old female patient with postoperative recurrence of DF in the right thigh was included in the surgery alone cohort. The patient still recurred 17 months after surgery with clinical symptoms. The patient was transferred to the surgery plus anlotinib cohort for continued treatment. After preoperative use of anlotinib, the tumor was resected again, the vascular nerve was preserved, and MRI showed no recurrence 14 months after surgery. A typical case of surgery plus anlotinib cohort was shown in Figure 4: A 21-year-old female was diagnosed with DF of the right buttock. The patient visited our hospital for the first time and underwent a needle biopsy at our hospital to confirm the diagnosis. The tumor was resected after preoperative use of anlotinib. MRI showed no tumor recurrence at 27, 39 and 49 months after the operation.

### 3.3 Complications and toxicity

Six patients in the surgery alone cohort had surgical complications, including 1 case of wound infection, 3 cases of wound healing problems, and 2 cases of temporary iatrogenic nerve injury; 7 patients in the surgery plus anlotinib cohort had complications, including 2 cases of wound infection, 4 cases of wound healing problems, and 1 case of temporary iatrogenic nerve injury. There was no statistical difference in complications between the two cohorts. Wound infections were resolved by debridement, wound healing problems by prolonging healing time, and temporary iatrogenic nerve injuries were all recovered within 6 months by the use of trophic nerve drugs (Table 2). Major adverse events included hand-foot-skin syndrome (n = 12, 52.2%), hypertension (n = 10, 43.5%), fatigue (n = 10, 43.5%), paramenia (n = 8, 34.8%), vomiting (n = 7, 30.4%), general or local pain (n = 7, 30.4%), proteinuria (n = 5, 21.7%), oral pain (n = 4, 17.4%), hemorrhage (n = 3, 13.0%), dizziness headache (n = 2, 8.7%). Paramenia was present in 8 female patients, accounting for 61.5% of female patients. These adverse events were generally grade 1 to 2, and only two patients had grade 3 adverse events (hypertension and hand-foot-skin syndrome), which were well controlled with symptomatic treatment or reduction in drug dose. None of the patients experienced grade 4 adverse events or discontinued the anlotinib because of side effects of the drug, which were considered tolerable by the patients (Table 3).

**TABLE 3:** Adverse events of anlotinib treatment.

Adverse events	n (%)	Grade 1	Grade 2	Grade 3
Hand-foot-skin syndrome	12 (52.2)	6	5	1
Hypertension	10 (43.5)	6	4	1
Fatigue	10 (43.5)	3	7	0
Paramenia	8 (34.8)	6	2	0
Vomiting	7 (30.4)	2	5	0
General or local pain	7 (30.4)	3	4	0
Proteinuria	5 (21.7)	5	0	0
Oral pain	4 (17.4)	2	2	0
Hemorrhage	3 (13.0)	2	1	0
Dizziness headache	2 (8.7)	1	1	0

## 4 Discussion

The present retrospective study described and analyzed the data from a series of patients with resectable extremity DF. DF of the extremities was usually located adjacent to neurovascular structures (as shown in Figure 4), and there was a greater risk of surgically injured vascular nerves compared to other areas, and more attention should be paid to such tumors. Therefore, patients with DF of the extremities were specifically included in this study. The primary objective of this study was to evaluate the tumor local recurrence rate of surgery combined with anlotinib in treating resectable extremity DF, and simultaneously, to evaluate the side effects of anlotinib.

The clinical management of DF remains challenging, and surgical resection of the tumor has previously been the standard primary treatment modality; however, in recent years, a shift to a more conservative management model has been introduced. A recent consensus reached by the DF Working Group suggests that aggressive treatment is recommended only in case of persistent progression, given the benign character and only local aggressiveness of the disease (2020). Clinical symptoms are incompletely associated with DF progression; some stable DF may be accompanied by clinical symptoms, while some progressive DF may have no clinical symptoms (Gronchi et al., 2014). Surgery may be considered if expected surgical morbidities are limited (Penel et al., 2021). Therefore, we focused on patients with clinical symptoms (mainly including pain, functional limitation, and compression symptoms), as an inclusion criterion to assess the efficacy and toxicity of surgery combined with anlotinib in the treatment of resectable extremity DF.

Many studies have shown that 23%–77% of tumors still have local recurrence after wide surgical resection (Pritchard et al., 1996; Vora et al., 2021; Mandel et al., 2022; Prendergast et al., 2022). The location of the tumor seems to have a major impact on local recurrence, with DF located in the extremities having recurrence rates of even as high as 80% (Salas et al., 2011; Lehnhardt et al., 2023). Similar to our findings, the postoperative local recurrence rate reached 72.0% in the surgery group alone at the last follow-up. With the use of combination therapies, including low-dose or conventional chemotherapy, NSAIDs, and TKIs, local recurrence rates have improved over the past few decades and have reportedly dropped to 17%–30% (Merchant et al., 1999; Shkalim Zemer et al., 2017; Wirth et al., 2018; Gronchi and Jones, 2019; Mikhael et al., 2022; Riedel and Agulnik, 2022; Tsukamoto et al., 2023). Although the RFS rate at the last follow-up in the surgery plus anlotinib cohort of this study was 34.8%, which was slightly higher than that reported in the literatures, there was a significant decrease compared to 72.0% in the surgery alone cohort. Meanwhile, the 3-year RFS rate was 72.6% in the surgery plus anlotinib cohort and 37.7% in the surgery alone cohort, and Kaplan-Meier analysis demonstrated that the RFS rate within the surgery plus anlotinib cohort exceeded that of the surgery alone cohort, with statistical significance (*p* = 0.022). The results showed that surgery combined with anlotinib could significantly reduce the local recurrence rate compared with surgery alone in the treatment of DF. While the local recurrence rate was effectively controlled, the MSTS score of patients in the surgery plus anlotinib cohort was significantly increased and the VAS score was significantly decreased compared with the surgery alone cohort, indicating that the clinical symptoms of patients in the surgery alone cohort were significantly improved.

Resection margins of the tumor are less important than maintaining function for the patient and do not have a significant impact on local recurrence (Wirth et al., 2018; Dürr et al., 2020). Unless the tumor was adjacent to the neurovascular bundle, the surgical procedure should aim for extensive resection. For cases with severe complications due to adjacent critical structures or after extensive resection, marginal resection was selected (as shown in Figures 3, 4). In a retrospective study of 426 patients diagnosed with DF, surgical margins (R2 v R0/R1) were found to have a significant impact on progression-free survival (PFS), but R0 v R1 did not (Salas et al., 2011). All surgical patients included in our study achieved R0 or R1 resection, and marginal resection of tumors adjacent to vascular nerves could be regarded as R1 resection, and those who could not reach R1 resection were considered unresectable lesions. Therefore, we did not include surgical margin classification as a study object in this study. Because the patients were compared between two different periods, although tumor resection was performed by the same surgeon in both cohorts, the surgical technique improved over time, which may also be one of the reasons for the decrease in local recurrence rate.

Recently, a phase III, randomized, placebo-controlled trial explored the efficacy of nirogacestat, a γ-secretase inhibitor, in adult patients with progressive DT (Gounder et al., 2023). The investigators randomly assigned 142 patients with desmoid tumors to receive either nirogacestat or placebo, and PFS was the primary endpoint. The study showed a significant PFS benefit with nigalrestat compared with placebo (hazard ratio for death or disease progression, 0.29; *p* < 0.001). The proportion of patients with objective response was significantly higher in the nirogacestat group than in the placebo group (41% vs. 8%; *p* < 0.001); the proportion of patients with complete response was 7% and 0%, respectively. Ninety-five percent of common adverse events with nirogacestat were grade 1 or 2, including diarrhea (84% of patients), nausea (54%), and fatigue (51%). Because of this study, nirogacestat was approved by the US Food and Drug Administration for the treatment of desmoid tumors. TKIs, as one of the systemic therapies, are effective in the treatment of DF, with 6-month PFS ranging from 65% to 96%, and the adverse event rates of grades 3 and 4 ranged from 0% to 15% and 0%–3%, respectively (Gounder et al., 2011; Kasper et al., 2017b; Agresta et al., 2018; Zheng et al., 2020). Imatinib was the first TKI used to treat DF, with a disease control rate of 78%–92% (Penel et al., 2011; Kasper et al., 2017b). In a double-blind phase III trial investigated by Gounder MM et al. (Gounder et al., 2018), 87 patients with DF received either sorafenib or matching placebo. With a median surveillance of 27 months, the 2-year PFS rate reached 81% and the median time to objective response was 9.6 months, both superior to placebo group. This clinical trial found that sorafenib significantly prolonged PFS with mild to moderate side effects, mainly including rash, fatigue, and hypertension events. Anlotinib is a novel multi-target TKI that inhibits tumor proliferation and angiogenesis with disease control rates and toxicity similar to sorafenib (Gounder et al., 2018; Shen et al., 2018; Tsukamoto et al., 2023). Zheng et al. (2020) retrospectively investigated the clinical data of 21 patients with extremity DF treated with anlotinib. 38.1% of the patients had partial response, 47.6% had stable disease, disease control rate up to 86.0%, and no patients had complete response. The results of the study showed that anlotinib was effective in DF with acceptable safety (mainly mild to moderate side effects) and significantly slowed disease progression (Zheng et al., 2020). In our study, the side effects of anlotinib were mainly mild to moderate and resolved by adjusting the drug and/or symptomatic treatment, and no drug withdrawal due to side effects occurred. Safety and side effects were controllable and similar to those reported in the literature. Furthermore, we used a lower dose (starting at 8 mg/day) compared to the dose used for soft tissue sarcomas (Chi et al., 2018), which theoretically would have lower side effects. In summary, escalation from less side effects to stepped therapy with more toxic agents is recommended for DF that requires medical treatment.

Additionally, we acknowledge that our study has several limitations. First, this study was retrospective and at risk of selection bias and recall bias. Second, owing to the limited number of patients in the study, there was a risk of Type II error. Third, there were few and incomplete data of patients who underwent surgery combined with radiotherapy or chemotherapy in our hospital, and no efficacy comparison was performed, but according to the results in the literature as a reference basis. Forth, the follow-up period of this study was relatively short, and the duration of anlotinib administration was uncertain, and some side effects of long-term efficacy could not be observed. At the same time, the follow-up time was different between the two cohorts, which may also cause bias, and further follow-up observation was required. Finally, the study excluded patients with abdominal or trunk lesions, and the sample size became limited with selection bias. Therefore, future studies including patients with abdominal or trunk lesions are warranted. These limitations should be taken into account when analyzing our findings.

## 5 Conclusion

In summary, in this retrospective study, surgery combined with anlotinib appears to be effective in controlling local recurrence in patients with resectable DF of the extremities, and side effects were acceptable. Additionally, the level of evidence in this study is observational and retrospective and that prospective randomized clinical trials with adequate power are needed to validate the therapeutic efficacy of surgery combined with anlotinib in resectable DF of the extremities.

## Data Availability

The raw data supporting the conclusion of this article will be made available by the authors, without undue reservation.
